# Risk factors for 90-day readmission in veterans with inflammatory bowel disease—Does post-discharge follow-up matter?

**DOI:** 10.1186/s40779-018-0153-x

**Published:** 2018-02-08

**Authors:** Ashish Malhotra, Parkpoom Phatharacharukul, Charat Thongprayoon

**Affiliations:** 1 Center of Innovation, Minneapolis VA Health Care System, Minneapolis, MN 55147 USA; 2 Division of Gastroenterology, Department of Medicine, Minneapolis VA Medical Center, Minneapolis, MN 55147 USA; 3 Department of Medicine, University of Minnesota School of Medicine, Minneapolis, MN 55147 USA; 4 Department of Internal Medicine, Bassett Medical Center, Cooperstown, NY 13326 USA

**Keywords:** Inflammatory bowel disease, Readmission rates, Veterans affairs

## Abstract

**Background:**

Repeat hospitalizations in veterans with inflammatory bowel disease (IBD) are understudied. The early readmission rate and potentially modifiable risk-factors for 90-day readmission in veterans with IBD were studied to avert avoidable readmissions.

**Methods:**

A retrospective cohort study was conducted using the data from veterans who were admitted to the Minneapolis VA Medical Center (MVMC) between January 1, 2007, and December 31, 2013, for an IBD-related problem. All-cause readmissions within 30 and 90 days were recorded to calculate early readmission rates. The multivariate logistic regression was used to identify the potential risk factors for 90-day readmission.

**Results:**

There were 130 unique patients (56.9% with Crohn’s disease and 43.1% with ulcerative colitis) with 202 IBD-related index admissions. The mean age at the time of index admission was 59.8 ± 15.2 years. The median time to re-hospitalization was 26 days (IQR 10-49), with 30- and 90-day readmission rates of 17.3% (35/202) and 29.2% (59/202), respectively. Reasons for all-cause readmission were IBD-related (71.2%), scheduled surgery (3.4%) and non-gastrointestinal causes (25.4%). The following reasons were independently associated with 90-day readmission: Crohn’s disease (*OR* 3.90; 95% CI 1.82-8.90), use of antidepressants (*OR* 2.19; 95% CI 1.12-4.32), and lack of follow-up within 90 days with a primary care physician (PCP) (*OR* 2.63; 95% CI 1.32-5.26) or a gastroenterologist (GI) (*OR* 2.44; 95% CI 1.20-5.00). 51.0% and 49.0% of patients had documentation of a recommended outpatient follow-up with PCP and/or GI, respectively.

**Conclusions:**

Early readmission in IBD is common. Independent risk factors for 90-day readmission included Crohn’s disease, use of antidepressants and lack of follow-up visit with PCP or GI. Further research is required to determine if the appropriate timing of post-discharge follow-up can reduce IBD readmissions.

## Background

Hospital readmission rate has been proposed as an important quality metric because such admissions are prevalent, expensive [1] and potentially avoidable [2]. Readmitted patients are 55% more likely to have a quality-of-care problem such as inadequate discharge instructions or outpatient follow-up scheduling [3], and repeat hospitalizations can be reduced by the intensive discharge planning of high-risk patients [3]. Medicare is increasingly instituting financial penalties for higher-than-expected readmission rates [4]. Consequently, it is imperative that organizations examine and implement strategies to avert avoidable readmissions.

Patients with chronic diseases are particularly prone to early readmission. Inflammatory bowel diseases (IBD) are a group of chronic inflammatory illnesses with a remitting and relapsing course that may result in considerable morbidity and high medical costs secondary to repeated hospitalizations [5]. Presently, there is a lack of data on the predictors of early readmission in veterans with IBD. In other chronic illnesses such as heart failure, intensive outpatient monitoring of patients after hospital discharge significantly reduces unplanned hospital admissions [6], and outpatient follow-up after hospitalization has become a routine part of their discharge planning. However, the impact on readmission rates of a post-discharge visit with a primary care provider (PCP) or a gastroenterologist (GI) has not been studied in IBD patients. In the present study, we examined the predictors of re-hospitalization in veterans with IBD, with attention to the hypothesis that an appropriate follow-up visit with a GI and/or PCP would reduce IBD-related readmissions.

## Methods

A retrospective cohort study was conducted using the data of adult patients who were admitted to Minneapolis VA Medical Center (MVMC), a tertiary-care referral center in Minneapolis, MN, between January 1, 2007, and December 31, 2013, for an IBD-related problem. Ethics approval was granted for the chart review by the Institutional Review Board at the MVMC (#4552-A).

IBD diagnosis was based on the international classification of diseases, Ninth Revision codes for ulcerative colitis (UC) or Crohn’s disease (CD) (ICD codes 556.X and 555.X, respectively) and documentation of IBD in the medical records by a physician. Patients with a non-elective hospitalization due to symptomatic IBD were included in the study. All patients were followed for 90 days after index hospitalization, and re-hospitalization admissions were defined as admissions (both IBD-related and non-IBD-related) that occurred within 90 days from the discharge date of the index hospitalization. Patients with multiple admissions had each admission separately considered if there were more than 90 days between admissions. Therefore, a patient could be counted twice provided that their admissions occurred at a minimum of 90 days from each other.

The following data were extracted from the electronic medical record: 1) patient demographics: age, gender, and race; 2) IBD history: IBD type (UC or CD), duration of IBD, previous surgical history, and medication regimen for IBD prior to hospital admission; 3) non-IBD history: comorbid psychiatric diagnoses and psychiatric medications such as anxiolytic drugs, anti-depressants, and anti-psychotics; 4) narcotic use prior to hospital admission; 5) hospitalization data: chief complaint, and principal diagnosis at discharge, intensive care unit (ICU) stay/transfer, duration of ICU stay, reason for ICU stay/transfer, surgical procedure(s) during hospitalization, nosocomial complications such as infection or thromboembolism, and length of stay; 6) discharge information: change in medication regimen for IBD, benzodiazepine prescription at discharge, narcotics prescription at discharge, and discharge destination (home or nursing facility); and 7) follow-up information: recommendation regarding follow-up appointment with a PCP and GI in discharge summary and/or discharge nurse note, patients’ attendance of follow-up appointment with a PCP and GI, and the duration between patients’ discharge date and the date they were seen by a PCP and/or GI.

The mean and standard deviation of the distribution described the continuous variables unless the results were highly skewed, in which case they were expressed using the median and interquartile range (IQR). In addition to basic descriptive statistics, univariate analyses were used to evaluate the association between readmissions and patient characteristics. Variables with a *P*-value of less than 0.25 in these simple logistic regression analyses were entered into multiple logistic models. In these models, factors not significant at the 0.05 level were removed. Statistical analyses were carried out using Version 10. SAS Institute Inc., Cary, NC, 1989-2007.

## Results

There were 130 unique patients accounting for 202 IBD-related index admissions during the study period. Table 1 lists baseline patient characteristics for the study cohort. Overall, 74 patients (56.9%) had CD, and 56 (43.1%) had UC. The mean age at the time of index admission was 59.8 ± 15.2 years. There were 35 readmissions within 30 days of the 202 index admissions yielding a rate of 17.3% and 59 readmissions within 90 days for a rate of 29.2%. The 30- and 90-day readmission rates were 6.5 and 4.1 per 1000 patient days, respectively. Out of 130 unique patients, 94 patients were admitted once, 20 were admitted twice, 7 were admitted 3 times, and 9 were admitted ≥ 4 times. Reasons for all-cause readmission within 90 days were for IBD-related problems (*n* = 144, 71.2%), scheduled surgery (*n* = 7, 3.4%), and non-gastrointestinal conditions (*n* = 51, 25.4%). The median time to 90-day rehospitalization was 26 days (IQR 10-49).

**Table 1:** Baseline characteristics of 130 unique IBD patients admitted to MVMC between 2007 and 2013 (*n* = 130)

Item	Value
Age (year, *x* ± *s)*	59.8 ± 15.2
Male (*n* (%))	125 (96.2)
Caucasian (*n* (%))	118 (90.8)
History of psychiatric disorder	66 (50.8)
Type of IBD (*n* (%))	
Crohn’s disease	74 (56.9)
Ulcerative colitis	56 (43.1)
Duration of IBD (year, median(IQR))	15 (5.0-30.3)
History of bowel resection (*n* (%))	75 (57.7)
IBD medication prior to admission (*n* (%))	
5-ASA	45 (34.6)
Immunomodulator (AZA/6-MP or MTX)	27 (20.8)
Anti-TNF	12 (9.2)
Steroids	25 (19.2)
Patient who has not been on IBD medication prior to admission (*n* (%))	37 (28.5)

*MVMC* Minneapolis VA Medical Center, *IBD* inflammatory bowel disease, *IQR* interquartile range, *ASA* aminosalicylic acid, *AZA* azathioprine, *6-MP* 6 Mercaptopurine, *MTX* methotrexate, *TNF* tumor necrosis factor

### Predictors of 90-day readmission

In a univariate analysis of various factors and 90-day rehospitalization, the presence of concomitant psychiatric disorder and CD (versus UC) were found to be significant (*P* < 0.05). Additionally, of all the psychiatric medications, the use of antidepressants prior to the index admission was significantly more common (55.9% vs. 32.9%, *P* < 0.01) in patients with readmissions. Lastly, changes in IBD medications during index hospitalization and discharge to home without follow-up appointment with a PCP or a GI within 90 days of discharge were also significant at the 0.05 level (Table 2).

**Table 2:** Univariate analysis of factors associated with 90-day readmission in veterans with IBD

Item	No readmission	90-day readmission	*P* value
Age (year, *x* ± *s)*	59.2 ± 15.6	61.4 ± 13.1	0.31
Male (*n* (%))	138 (96.5)	59 (100)	0.15
Caucasian (*n* (%))	128 (89.5)	57 (96.6)	0.10
History of psychiatric disorder (*n* (%))	74 (51.7)	42 (71.2)	0.01
Type of IBD (*n* (%))			< 0.001
Crohn’s disease	78 (54.5)	48 (81.4)	
Ulcerative colitis	65 (45.5)	11 (18.6)	
Duration of IBD (year, median(IQR))	16 (7-30)	16 (3-34)	0.84
History of bowel resection (*n* (%))	95 (66.4)	39 (66.1)	0.96
IBD medication prior to admission (*n* (%))			
5-ASA	51 (35.7)	15 (25.4)	0.16
Immunomodulator (AZA/6-MP or MTX)	29 (20.3)	15 (25.4)	0.42
Anti-TNF	18 (12.6)	6 (10.2)	0.63
Steroids	25 (17.5)	15 (25.4)	0.20
Psychiatric/pain medication prior to admission (*n* (%))			
Benzodiazepine	24 (16.8)	9 (15.3)	0.79
Narcotics	57 (39.9)	28 (47.5)	0.32
Antidepressant	47 (32.9)	33 (55.9)	0.002
Medication at discharge (*n* (%))			
Change in IBD medication at discharge	32 (22.4)	23 (39)	0.02
Benzodiazepine at discharge	22 (15.4)	8 (13.6)	0.74
Narcotics at discharge	75 (52.4)	29 (49.2)	0.67
Discharge to home (*n* (%))	135 (94.4)	51 (86.4)	0.06
Complication during hospitalization (*n* (%))			
Surgical procedures during admission	28 (19.6)	10 (16.9)	0.66
ICU stay	6 (4.2)	3 (5.1)	0.78
Nosocomial infection	6 (4.2)	2 (3.4)	0.79
Venous thromboembolism	1 (0.7)	1 (1.7)	0.52
Length of hospitalization (day, median (IQR))	4 (2-6)	4 (2-8)	0.84
Follow-up after hospitalization (*n* (%))			
Mention of follow-up with PCP in discharge summary/nurse note	71 (49.7)	32 (54.2)	0.55
Follow-up with PCP clinic within 90 days	74 (51.7)	16 (27.1)	0.001
Mention of follow-up with GI in discharge summary/nurse note	67 (46.9)	31 (52.5)	0.46
Follow-up in GI clinic within 90 days	67 (46.9)	18 (30.5)	0.03

*IBD* inflammatory bowel disease, *IQR* interquartile range, *PCP* primary care physician, *TNF* tumor necrosis factor, *ICU* Intensive care unit, *GI* gastroenterologist

In a multivariate model, CD, the use of antidepressants, and lack of follow-up with a PCP and a GI within 90 days were independently positively associated with 90-day readmission (Table 3).

**Table 3:** Risk factors independently associated with readmissions in IBD patients within 90 days of discharge

Variables	*OR*	95% CI	*P* value
CD (compared to UC)	3.90	1.82-8.90	< 0.001
Use of antidepressants prior to admission	2.19	1.12-4.32	0.02
Lack of follow-up with PCP within 90 days after discharge	2.63	1.32-5.26	0.006
Lack of follow-up with GI within 90 days after discharge	2.44	1.20-5.00	0.01

*IBD* inflammatory bowel disease, *CD* Crohn’s disease, *UC* ulcerative colitis, *PCP* primary care physician, *GI* gastroenterologist

### Follow-up visits after index admission

51.0% (*n* = 103) and 49.0% (*n* = 99) of patients had documentation of a recommended outpatient follow-up with a PCP and/or GI, respectively. Ninety patients (44.6%) had a follow-up visit with a PCP only, 85 (42.1%) had a visit with a GI only, and 27 (13.3%) had visits with both within 90 days of discharge. For the 90 days following discharge for the 202 index admissions, there was no correlation between readmission rate and the time elapsing between discharge and follow-up with a PCP: 22 days (IQR: 8.8-43.3) with no readmission and 17.5 days (IQR: 8.3-33.8) with readmission (*P* = 0.35). The median duration of a follow-up with a GI was longer in patients with no readmission (34 days (IQR: 17-55)) when compared with patients with readmission (14 days (IQR: 10.3-26.5)) (*P* = 0.004). A factor independently associated with not attending a follow-up visit with a PCP and/or a GI was the lack of documentation of a recommended follow-up with PCPs (*P* = 0.02) and GIs (*P* < 0.01).

## Discussion

The rationale of this retrospective study of veterans with IBD is that the understanding of the factors related to readmission may enhance our ability to reduce frequent readmissions in this group of patients. Several findings deserve emphasis. First, the 30-day and 90-day readmission rates were high at 17.3 and 29.2%, respectively. Second, we found that the risk factors for 90-day readmission in veterans with IBD were CD (versus UC), the use of antidepressants, and the lack of a follow-up visit with a primary care provider and/or a GI patient after discharge. Third, the lack of documentation of a recommended outpatient follow-up upon discharge and not being on any narcotics or benzodiazepines were associated with the lack of outpatient follow-up after discharge.

Prior studies in non-Veteran Affairs (VA) patients found the 90-day readmission rates for IBD patients to range from 20.5%–35.1%, which is similar to our findings [7, 8]. Our finding of higher readmission rates among veterans with CD is similar to a previous study [9], a finding that reflects the more aggressive behavior and higher frequency of poor outcomes in CD than in UC [10–12]. Some of the factors leading to more complications in CD versus UC include the potentially greater extent of gut that may be involved [13], more frequent structuring or penetrating complications, [13] and disease recurrence after resection of all grossly involved areas.

Several previous studies have investigated the relationship between IBD and psychiatric disease in non-VA patients. Depression is common and linked to more severe disease activity and a poorer quality of life [14]. Allegretti et al. [7] found that depression is a risk factor for readmission in IBD patients, similar to our study. There are multiple potential explanations for this association. Psychiatric comorbidities can affect systemic inflammation and thus trigger disease activity [15, 16], and coping mechanisms in IBD patients with a psychiatric disease might be poorer than those without psychiatric disorders increasing the frequency of visits to urgent care centers. Lastly, patients with IBD have been found to have higher perceptions of pain, which has been linked to depression [17]. Our cohort demonstrates a positive association between the use of antidepressants and increased re-hospitalization rates, confirming that concomitant clinical depression leads to increased healthcare use in IBD patients.

Various studies conducted on reducing readmission rates in patients with chronic diseases suggest that closer outpatient follow-up may reduce rehospitalizations [18, 19]. Our study also showed that an outpatient visit was associated with reduced readmission rates. The major factor correlating with an outpatient visit was the scheduling of the next appointment upon hospital discharge, a finding that underlines the importance of attention to detail at the time of discharge. The appropriate time for the outpatient visit is not clear. One might postulate that earlier might be better than later to combat the problems leading to readmission. However, a prior study showed that if a gastroenterology follow-up appointment was scheduled after, rather than before, the first 22 days after discharge, the patient was less likely to be readmitted within 30 days [20]. We also found that the mean duration between discharge and follow-up with either a PCP or a GI was shorter for readmitted subjects (17.5 and 14 days) than for those who were not readmitted (22 and 34 days) (*P* value = 0.35 and 0.004, respectively). This observation should not be misconstrued to indicate that an early follow-up visit with a GI causes early readmission but rather that sicker patients were provided with an early GI follow-up visit. As discussed, a post-discharge appointment with a GI was associated with a reduced rate of readmission over the 90-day follow-up period. It seems likely that this finding is explained by a tendency to provide more rapid outpatient follow-up to the patients with the most severe disease, and a far more complex study than the present retrospective analysis will be required to ascertain the appropriate timing of the outpatient follow-up visit. The period immediately following hospital discharge is a vulnerable time for IBD patients, since during this time, they adjust to new drugs, recover from an acute illness and cope with the challenges of IBD. Although randomized trials in the IBD population to reduce readmissions are lacking, data from other chronic diseases have shown that the use of integrated multidisciplinary clinics to address the social and psychological burdens of chronic illness and the use of nurse case managers or “transitions coach” to improve cross-site communication, encouraging patients to take a more active role in their care by providing guidance, can reduce readmissions and improve outpatient follow-up [21, 22]. A previous study in VA patients done by our group demonstrated that IBD patients were more likely to have significant changes in their medication profiles upon discharge from the hospital, thus highlighting the need for transitional care interventions [23].

Our study limitations merit attention. We relied on available clinical and administrative data to assess and account for differences in illness severity at the patient or hospital level. Thus, residual confounding cannot be ruled out. We were also unable to ascertain the necessity of hospitalization or the criteria used to decide to readmit patients. Rehospitalization of the veteran at a different institution would result in an underestimation of the rate of readmission. Finally, the generalizability of our study may be limited by the differences between veteran and non-veteran patients including social, psychological, and medical factors. It is also possible that differences between patients at various VA facilities could limit the generalizability of our findings to all VA institutions.

## Conclusion

Early readmission in IBD is common. Independent risk factors for 90-day readmission included CD, the use of antidepressants and the lack of follow-up visit with a PCP or a GI. Although further research is required to assess the precise timing and role of outpatient follow-up in reducing IBD readmissions, this paper highlights the need to study transitional care interventions to reduce rehospitalization rates in veterans with IBD.

## Abbreviations

CD: Crohn’s disease
GI: Gastroenterologist
IBD: Inflammatory bowel disease
ICU: Intensive care unit
IQR: Interquartile range
MVMC: Minneapolis VA Medical Center
PCP: Primary care physician
TNF: Tumor necrosis factor
UC: Ulcerative colitis
VA: Veteran Affairs
